# MAMGN-HTI: A Graph Neural Network Model with Metapath and Attention Mechanisms for Hyperthyroidism Herb–Target Interaction Prediction

**DOI:** 10.3390/bioengineering12101085

**Published:** 2025-10-05

**Authors:** Yanqin Zhou, Xiaona Yang, Ru Lv, Xufeng Lang, Yao Zhu, Zuojian Zhou, Kankan She

**Affiliations:** 1 School of Artificial Intelligence and Information Technology, Nanjing University of Chinese Medicine, Nanjing 210023, China; zhouyanqin@njucm.edu.cn (Y.Z.); yangxiaona0714@163.com (X.Y.); lvru@njucm.edu.cn (R.L.); langxufeng@njucm.edu.cn (X.L.); 2 Jiangsu Province Engineering Research Center of TCM Intelligence Health Service, Nanjing University of Chinese Medicine, Nanjing 210023, China; 3 Zhou Zhongying Studio of National Medical Master, Nanjing University of Chinese Medicine, Nanjing 210023, China; zhuyao@njucm.edu.cn

**Keywords:** herb–target prediction, graph neural network, metapath, attention mechanism, hyperthyroidism

## Abstract

The accurate prediction of herb–target interactions is essential for the modernization of traditional Chinese medicine (TCM) and the advancement of drug discovery. Nonetheless, the inherent complexity of herbal compositions and diversity of molecular targets render experimental validation both time-consuming and labor-intensive. We propose a graph neural network model, MAMGN-HTI, which integrates metapaths with attention mechanisms. A heterogeneous graph consisting of herbs, efficacies, ingredients, and targets is constructed, where semantic metapaths capture latent relationships among nodes. An attention mechanism is employed to dynamically assign weights, thereby emphasizing the most informative metapaths. In addition, ResGCN and DenseGCN architectures are combined with cross-layer skip connections to improve feature propagation and enable effective feature reuse. Experiments show that MAMGN-HTI outperforms several state-of-the-art methods across multiple metrics, exhibiting superior accuracy, robustness, and generalizability in HTI prediction and candidate drug screening. Validation against literature and databases further confirms the model’s predictive reliability. The model also successfully identified herbs with potential therapeutic effects for hyperthyroidism, including Vinegar-processed Bupleuri Radix (Cu Chaihu), Prunellae Spica (Xiakucao), and Processed Cyperi Rhizoma (Zhi Xiangfu). MAMGN-HTI provides a reliable computational framework and theoretical foundation for applying TCM in hyperthyroidism treatment, providing mechanistic insights while improving research efficiency and resource utilization.

## 1. Introduction

Hyperthyroidism is a common and complex endocrine disorder that has attracted widespread attention due to its high prevalence and multifactorial pathogenesis [1]. Although modern medicine has made substantial progress in the treatment of hyperthyroidism, challenges remain in achieving long-term regulation and providing comprehensive, system-level intervention. Rooted in the principles of holism and syndrome differentiation, traditional Chinese medicine (TCM) has demonstrated distinct advantages in the integrated management of hyperthyroidism. Professor Zhou Zhongying, the first National Master of Traditional Chinese Medicine, dedicated his career to the study of TCM pathomechanisms and clinical applications. He proposed the theory of the “*Thirteen Pathomechanisms*” [2,3], which emphasizes causal linkages among complex pathological factors and established a novel paradigm for pathomechanism-based syndrome differentiation in TCM, thereby providing a theoretical foundation for the clinical classification and treatment of hyperthyroidism. With the advancement of TCM modernization and precision medicine, there is an urgent need to apply modern computational approaches to elucidate herb–disease target interactions, thereby strengthening the scientific foundation and mechanistic interpretability of TCM therapies.

This study aims to investigate the interactions between core herbs and targets in hyperthyroidism, based on Professor Zhou Zhongying’s theory of pathomechanism-based syndrome differentiation. Clinical records of hyperthyroidism cases treated by Professor Zhou were analyzed and integrated with the authors’ previous work on the prediction of hyperthyroidism-related pathomechanism–herb communities [4]. To this end, we developed a graph neural network model that incorporates metapaths and attention mechanisms and systematically constructed a heterogeneous herb–target association network. Through hierarchical feature extraction and relational modeling, the model enables accurate prediction of herb–target interactions, thereby revealing the potential pharmacological mechanisms of traditional Chinese medicine in the treatment of hyperthyroidism. This computational framework provides data-driven support for precision herbal therapy in TCM and offers novel perspectives and methodologies for advancing hyperthyroidism treatment.

In recent years, advances in computational pharmacology and graph learning techniques have emerged as powerful approaches for elucidating herb–target relationships. Gan et al. [5] proposed a method for predicting traditional Chinese medicine (TCM)–symptom treatment combinations based on the human protein–protein interaction network. Wang et al. [6] developed the DTI-BGCGCN model, which integrates bipartite graphs with clustered graph convolutional networks to predict targets for both modern drugs and herbal medicines. Hu et al. [7] designed a dual-channel hypergraph convolutional network, HGHDA, to embed herbal ingredients and target proteins into a low-dimensional space while preserving similarity features. Qiu et al. [8] introduced the LSTM-SAGDTA model, which combines SeqVec embeddings with graph neural networks and employs a self-attention mechanism to enhance prediction accuracy. Qu et al. [9] constructed an end-to-end graph neural network framework that integrates heterogeneous network data to achieve representation learning for drug–target prediction. Collectively, these studies have laid an important foundation for applying graph learning models to systematically investigate herb–target interactions in TCM research.

Graph Neural Networks (GNNs), a deep learning model designed to learn node and edge representations from graph-structured data, have been widely applied to tasks involving molecular and biological networks due to their outstanding ability to model graph-structured data [10]. Nevertheless, conventional GNNs encounter notable challenges when applied to heterogeneous graph structures, particularly in addressing sample imbalance and the problem of over-smoothing. Yang et al. [11] proposed IS-GNN, which integrates structural and homogeneity information to effectively mitigate performance degradation in heterogeneous graphs. Wang et al. [12] introduced NLA-GNN, a non-local aggregation framework that leverages attention mechanisms to incorporate long-range information. Li et al. [13] developed KNN-GNN, which utilizes a shared-subspace K-nearest neighbor strategy to improve classification performance across networks with varying levels of homogeneity. Building upon these studies, the present study introduces a multi-level message passing mechanism designed to optimize the representation of multiple node types—namely herbs, efficacies, ingredients, and targets—thereby strengthening the structural modeling capacity for herb–target interaction prediction.

Meta-paths are path sequences used to model specific semantic relationships in heterogeneous information networks and serve to uncover semantic correlations among nodes. Jongmin Park et al. [14] developed MIGTNet, a graph embedding model based on meta-path instances that employs hierarchical graph attention to enhance representation quality. Ma et al. [15] introduced SESIM, which enables self-supervised learning of meta-path structural information, thereby reducing dependence on labeled data. Lai et al. [16] proposed MIGP, which incorporates a learnable prompt mechanism during the pretraining phase to improve generalization in low-resource scenarios. In this study, meta-paths are utilized to explicitly model multi-hop semantic relationships among herbs, efficacies, ingredients, and targets, thereby strengthening global semantic representation and improving predictive performance.

Attention mechanism, a strategy that adaptively assigns weights to features or paths to highlight more informative signals, has been widely applied in modeling graph-structured data due to its efficient information filtering and weighting capabilities. Wu et al. [17] integrated multi-head attention into the protein function prediction model CFAGO, enabling the combination of protein–protein interaction (PPI) networks with protein features and effectively mitigating the over-smoothing problem. Liu et al. [18] proposed BAB-GSL, which enhances graph structural learning by leveraging attention mechanisms. Hu et al. [19] utilized temporal-guided attention in temporal knowledge graph reasoning to improve inference accuracy. In this study, attention mechanisms are employed to dynamically assign semantic weights to distinct meta-paths, thereby highlighting the contributions of key semantic pathways to herb–target associations while suppressing redundancy, ultimately improving the model’s generalization capacity and interpretability.

To address the challenges posed by the strong heterogeneity of traditional Chinese medicine (TCM) data, limited annotations, and the intricate nature of herb–target relationships, this study proposes MAMGN-HTI (Graph Neural Network with Metapath and Attention Mechanism for Prediction of Herb–Target Interactions), a graph neural network model for herb–target prediction. By integrating the cross-layer information propagation mechanisms of Residual Graph Convolutional Network (ResGCN) and Densely Connected Graph Convolutional Network (DenseGCN) with metapath and attention mechanisms, the model substantially improves the representation learning in heterogeneous graphs. ResGCN leverages residual connections to retain both initial and intermediate features, strengthening cross-layer information flow among herb, efficacy, ingredient, and target nodes while mitigating vanishing gradient issues. DenseGCN utilizes dense connections to maximize feature reuse, strengthen gradient flow, and improve representation capacity. The model further captures diverse semantic relationships among herbs, ingredients, targets, and efficacies in the heterogeneous graph through meta-paths, while the attention mechanism dynamically identifies and assigns weights to key meta-paths, thereby optimizing information propagation across nodes. Experimental results demonstrate that this model exhibits significant advantages in predicting herb–target interactions. Overall, MAMGN-HTI underscores the potential of GNN-based approaches in herb–target interaction prediction and provides novel computational strategies for advancing hyperthyroidism treatment research.

## 2. Materials and Methods

### 2.1. Preparation

**Figure 1:**
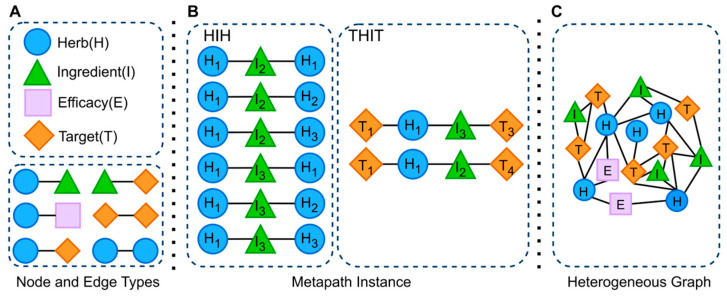
Overview of key terms in the preparatory work. (**A**) Node and edge types in the model, (**B**) two metapath instances, and (**C**) the constructed heterogeneous graph.

This section provides formal definitions of the terms used in this paper. A graphical illustration is presented in Figure 1 for better clarity.

#### 2.1.1. Heterogeneous Graph

A heterogeneous graph, which can represent multiple types of nodes and edges, is constructed with four entity types: Herb (H), Efficacy (E), Ingredient (I) and Target (T). In this graph, nodes correspond to distinct entity types, while edges encode their relationships, including herb–ingredient interactions (H-I), herb–efficacy interactions (H-E), ingredient–target interactions (I-T), herb–herb interactions (H-H), target–target interactions (T-T), and potential herb–target interactions (H-T). This heterogeneous graph effectively captures the diversity of entity types and the complexity of their relationships, thereby providing a robust framework for modeling the intricate task of herb–target prediction.

#### 2.1.2. Metapath

A metapath is a path schema that characterizes semantic relationships among nodes in a heterogeneous graph, with each metapath capturing a distinct type of semantic association. For example, the HIH metapath indicates that a specific ingredient is shared between two distinct herbs. A metapath instance refers to the concrete realization of a metapath schema within the graph structure.

#### 2.1.3. Metapath Instance

A metapath instance is defined as the concrete realization of a metapath within a heterogeneous graph, consisting of actual nodes and edges that conform to the definition of a specific metapath. It represents the explicit instantiation of the abstract metapath pattern. As illustrated in Figure 1B, for the metapath HIH, the herb node H_1_ exhibits six metapath instances (i.e., H_1_I_2_H_1_, H_1_I_2_H_2_, H_1_I_2_H_3_, H_1_I_3_H_1_, H_1_I_3_H_2_, and H_1_I_3_H_3_). Similarly, for the metapath THTI, the target node T_1_ is linked to two metapath instances (i.e., T_1_H_1_I_3_T_3_ and T_1_H_1_I_2_T_4_).

#### 2.1.4. Metapath Neighbor Node

A metapath neighbor node is defined as the collection of nodes within a heterogeneous graph that are connected to a given node via a specific metapath. This set encompasses both the node itself and all nodes reachable through the metapath. For instance, considering the metapath THTI, the metapath neighbor nodes of the target node T_1_ consist of all nodes accessible from T_1_ through the HTI path.

### 2.2. Methods

**Figure 2:**
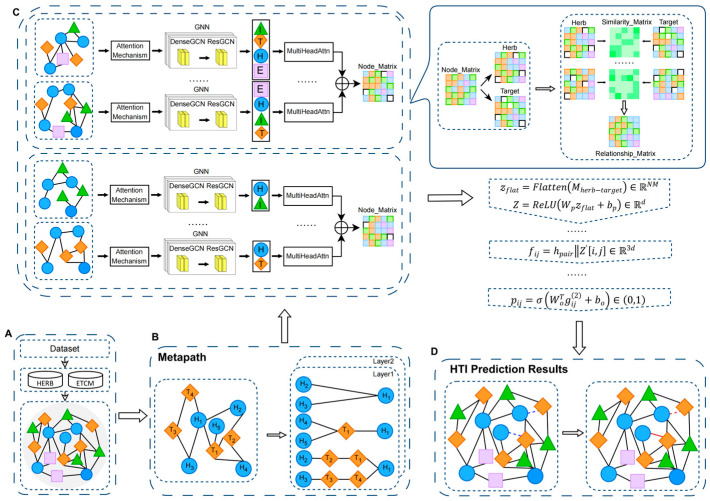
The MAMGN-HTI framework consists of dataset preparation and three main modules: data preparation (**A**), metapath construction (**B**), Herb–Target Representation Learning (**C**), and HTI Prediction (**D**). In panel (**D**), blue dashed lines indicate predicted pairs, red solid lines represent existing relationships, and red dashed lines indicate no relationship.

In this section, the details of MAMGN-HTI are introduced, including three key components: Metapath Construction module, Herb–Target Representation Learning module and HTI Prediction module. The overall framework of MAMGN-HTI is shown in Figure 2.

#### 2.2.1. Metapath Construction

In this module, heterogeneous node information is systematically integrated from multiple authoritative biomedical databases (e.g., TCMSP, HERB) to ensure data reliability and coverage. Based on these curated datasets, a heterogeneous graph is constructed with four domain-specific node types: herb (H), efficacy (E), ingredient (I), and target (T), where each node category is initialized with tailored feature representations. To effectively model the complex relationships between herbs and targets, a dual-channel representation learning framework is designed, which processes herb and target nodes independently to preserve their unique attributes. Within this framework, metapaths are formulated for herb and target nodes under carefully defined structural constraints. Through a trainable attention mechanism, the model dynamically assesses the semantic relevance of different metapaths. The cosine similarity between node features and target nodes in each metapath is first calculated as the preliminary weight, followed by the generation of normalized attention coefficients through a parameterized attention network, which jointly encode the path topology and node embedding. For herb nodes, metapaths are constrained to both originate and terminate at herb nodes, while target node metapaths follow the same principle. This design ensures that the generated metapaths highlight the structural semantics of target nodes while maintaining compatibility with conventional Graph Neural Networks. To balance semantic information capture and computational efficiency, the maximum metapath transmission length is limited to 5. This length effectively captures structural information between nodes while mitigating the computational complexity, memory overhead, and potential noise associated with longer paths.

Therefore, for the heterogeneous graph shown in Figure 1C, 10 metapaths (i.e., HH, HTH, HIH, HEH, HTTH, HTHTH, HIIH, HEHEH, HTITH, HITIH) are obtained for each herb node and 8 metapaths (i.e., TT, THT, TIT, THHT, TITIT, THTHT, THIHT, TIHIT) for each target node. These metapaths construct complex relationships between herbs and targets from both structural and semantic perspectives.

#### 2.2.2. Herb–Target Representation Learning Module

In this module, nodes and edges within the herb–target heterogeneous graph are embedded into vector representations to effectively capture their semantic attributes and relational dependencies. The procedure consists of the following steps:

Node Selection: A herb node is designated as the target node from the heterogeneous graph, while its associated efficacy nodes and target nodes are identified as source nodes.

Input Information Extraction: For the target node and its associated source nodes, the connecting edges together with their meta-relations are retrieved as inputs. An attention-based weighting mechanism is then applied to both nodes and edges based on their meta-relations, emphasizing the relative importance of different node and edge types.

(1) $$\alpha_{r,u\to h_{1}}=\frac{exp\left(LeakyReLU\left(a_{r}^{T}\left[W_{r}^{\left(l\right)}h_{u}^{\left(l-1\right)}||W_{r}^{\left(l\right)}h_{h_{1}}^{\left(l-1\right)}\right]\right)\right)}{\sum_{k\in N_{r}\left(h_{1}\right)}exp\left(LeakyReLU\left(a_{r}^{T}\left[W_{r}^{\left(l\right)}h_{k}^{\left(l-1\right)}||W_{r}^{\left(l\right)}h_{h_{1}}^{\left(l-1\right)}\right]\right)\right)}$$

$\alpha_{r,u\to h_{1}}$ represents the attention weight from node u to $h_{1}$ under relation r. $a_{r}$ denotes the attention weight vector for relation r, and || signifies the vector concatenation operation.

Node Representation Learning: The input node and edge features are leveraged to learn contextual representations of each node through multiple neural network layers. This process involves encoding node and edge features, followed by nonlinear transformations across layers to generate enriched node representations.

(2) $$m_{h_{1}}^{\left(l\right)}=\sum_{r\in R}\sum_{u\in N_{r}\left(h_{1}\right)}\alpha_{r,u\to h_{1}}\cdot \phi_{r}^{\left(l\right)}\left(h_{u}^{\left(l-1\right)},h_{h_{1}}^{\left(l-1\right)},e_{r}^{\left(l\right)}\right)$$

R denotes the set of all edge types in the graph. $N_{r}\left(h_{1}\right)$ represents the set of neighboring nodes connected to node $h_{1}$ via relation r. $h_{u}^{\left(l-1\right)}$ corresponds to the embedding representation of node u at layer l−1, while $h_{h_{1}}^{\left(l-1\right)}$ refers to the embedding representation of node $h_{1}$ at the same layer. Additionally, $e_{r}^{\left(l\right)}$ indicates the edge feature representation of relation r.

(3) $$h_{h_{1}}^{\left(l\right)}=\sigma \left(W^{\left(l\right)}\cdot m_{h_{1}}^{\left(l\right)}+b^{\left(l\right)}\right)$$

$h_{h_{1}}^{\left(l\right)}$ denotes the embedding representation of node v at layer l. Here, σ represents the nonlinear activation function, while $W^{\left(l\right)}$ and $b^{\left(l\right)}$ correspond to the learnable parameters at layer l.

Information Aggregation: During the learning process, the features of the source node are aggregated onto the target node $h_{1}$ by leveraging meta-relations and edge attributes, thereby updating and enriching the target node’s representation.

(4) $$h_{h_{1}}^{\left(l\right)}=Aggregate\left(\left\{m_{h_{1}}^{\left(l\right)}|r\in R\right\}\right)$$

Aggregate refers to the aggregation function.

This step captures the correlation and influence between the source node and the target node.

Iterative Update: Through successive operations of the multi-layer neural network, information from the source node is progressively propagated and aggregated onto the target node until the final representation of node $h_{1}$ is obtained.

(5) $$h_{h_{1}}^{\left(final\right)}=h_{h_{1}}^{\left(L\right)}$$

L refers to the number of layers in the GNN.

Cross-Hierarchical Attention Pooling: This mechanism is developed to integrate node representations across multiple layers in an L-layer Graph Neural Network (GNN). The final node representation is obtained by employing a multi-head attention mechanism to weight and fuse hierarchical features.

(6) $$h_{v}^{\left(final\right)}=MultiHeadAttn\left(\left[h_{v}^{\left(1\right)}⨁r_{\varphi }^{\left(1\right)},\cdots ,h_{v}^{\left(L\right)}⨁r_{\varphi }^{\left(L\right)}\right]\right)$$

Here, ⨁ denotes the vector concatenation operation, $r_{\varphi }^{\left(l\right)}$ represents the meta-relation embedding vector at layer l, and MultiHeadAttn denotes the multi-head attention mechanism. The computation formula is as follows:

(7) $${head}_{i}=softmax\left(\frac{Q_{i}K_{i}^{T}}{\sqrt{d}}\right)V_{i}$$

Here, $Q_{i}=W_{i}^{Q}\left[h_{v}^{\left(l\right)}⨁r_{\varphi }^{\left(l\right)}\right]$,$K_{i}=W_{i}^{K}\left[h_{v}^{\left(l\right)}⨁r_{\varphi }^{\left(l\right)}\right]$,$V_{i}=W_{i}^{V}h_{v}^{\left(l\right)}$. In this step, the final representations of all herb nodes, $h_{v}^{\left(final\right)}$, are automatically assembled into the herb node matrix $H_{herb}\in R^{N\times d}$, while the final representations of all target nodes, $h_{v}^{\left(final\right)}$, form the target node matrix $H_{target}\in R^{M\times d}$.

Heterogeneous Relation Matrix Construction: The attention-enhanced matrix generation process comprises two principal stages: computation of meta-relational attention weights and synthesis of the relation matrix.

Metarelational Attention Weight Generation:

(8) $$\beta_{\varphi }=softmax\left(W_{\varphi }\cdot \left[\mu_{\varphi }⨁\bar{h_{v}}\right]\right)$$

Here, $u_{\varphi }$ represents the predefined metarelational prototype, and $\bar{h_{v}}$ denotes the globally pooled contextual vector.

Attention-Enhanced Relation Matrix:

(9) $$M_{herb-target}=\sum_{\varphi \in \phi }\beta_{\varphi }\cdot \sigma \left(H_{herb}\cdot {\left(W_{\varphi }H_{target}\right)}^{T}\right)$$

Here, Φ represents the set of all relevant metarelations, and $W_{\varphi }$ denotes the relation-specific linear transformation matrix.

Through the aforementioned steps, node information within the Herb–Target heterogeneous graph is effectively embedded into continuous vector representations. These representations encapsulate the semantic information and relational patterns of the nodes, thereby enhancing the model’s capability to capture and predict herb–target interactions. This process provides a robust foundation for downstream prediction tasks.

To further enhance the efficiency of information propagation and the quality of node representation learning, this study proposes a heterogeneous graph neural network augmented with a skip connection mechanism. The study innovatively integrates ResGCN and DenseGCN into heterogeneous graph representation learning, optimizing model performance through improved cross-layer information propagation and mitigated information loss. By constructing a heterogeneous graph comprising four entity types—herb, efficacy, ingredient, and target—along with their associated relationships, the message-passing mechanism of the graph neural network effectively captures the complex interactions among diverse node and edge types in the heterogeneous graph. This framework substantially improves the performance of herb–target representation learning.

In shallow networks, DenseGCN adopts a dense connectivity scheme, where the output of each layer is concatenated with the outputs of all preceding layers. This approach enhances feature reuse and facilitates gradient propagation, rendering it particularly effective for modeling the complex and diverse relationships among entities in heterogeneous graphs. The dense connection mechanism in DenseGCN can be formally represented as follows:

(10) $$h_{i}^{\left(l+1\right)}=\sigma \left(W^{\left(l\right)}\cdot \left[h_{i}^{\left(0\right)}||h_{i}^{\left(1\right)}||\cdots ||h_{i}^{\left(l\right)}\right]+b^{\left(l\right)}\right)$$

Here, $h_{i}^{\left(0\right)}||h_{i}^{\left(1\right)}||\cdots ||h_{i}^{\left(l\right)}$ represents the concatenation of node features from layer 0 to layer l to form the new input; $W^{\left(l\right)}$ denotes the learnable weight matrix at layer l, which performs a linear transformation on the concatenated features; and $b^{\left(l\right)}$ is the bias term at layer l.

In deep networks, ResGCN incorporates a residual connection mechanism, where the input of each layer is directly added to its output. This ensures stable information flow from shallow to deeper layers, effectively mitigating issues such as information loss and vanishing gradients. The representation update formula for ResGCN is formally defined as follows:

(11) $$h_{i}^{\left(l+1\right)}=\sigma \left(\sum_{j\in N\left(i\right)}\frac{1}{c_{ij}}W^{\left(l\right)}h_{j}^{\left(l\right)}+h_{i}^{\left(l\right)}\right)$$

Here, $h_{i}^{\left(l\right)}$ represents the feature embedding of node i at layer l. $N\left(i\right)$ denotes the set of neighboring nodes for node i and $c_{ij}$ is the normalization coefficient for neighboring nodes. $W^{\left(l\right)}$ corresponds to the learnable weight matrix at layer l. The input to the residual connection, $h_{i}^{\left(l\right)}$ is directly added to the result of message aggregation.

By integrating ResGCN and DenseGCN, this study systematically incorporates the skip connection mechanism into heterogeneous graph representation learning. This approach significantly enhances the performance of herb–target representation learning and provides robust feature embeddings to support downstream target prediction tasks.

#### 2.2.3. HTI Prediction Module

In this module, a prediction framework leveraging node embedding and a fully connected network is proposed to predict the interactions between herbs and targets. This is achieved through multi-level feature transformation and nonlinear interaction modeling. The workflow proceeds as follows:

The heterogeneous relationship matrix $M_{herb-target}$, produced by the herb–target representation learning module, is flattened into a one-dimensional vector and subsequently projected into a high-dimensional feature space through a linear transformation layer.

(12) $$z_{flat}=Flatten\left(M_{herb-target}\right)\in R^{NM}$$

(13) $$Z=ReLU\left(W_{p}z_{flat}+b_{p}\right)\in R^{d}$$

Here, $W_{P}\in R^{d\times NM}$ denotes a learnable projection matrix, and $b_{p}\in R^{d}$ is the bias term. This step applies a nonlinear transformation to encode the sparse matrix association patterns into a dense global feature vector Z, effectively capturing the overall topological structure of the herb–target interactions.

Dual-Path Feature Fusion Mechanism: For each candidate herb–target pair $\left(h_{i},t_{j}\right)$, a dual-path feature fusion strategy is implemented, integrating both local semantic features and global association embeddings. The local semantic features are derived by extracting the embedding vectors of the herb node $h_{herb}^{i}\in R^{d}$ and the target node $h_{target}^{j}\in R^{d}$, which are concatenated to construct node-level features.

(14) $$h_{pair}=\left.h_{herb}^{i}\right\|h_{target}^{j}\in R^{2d}$$

The global association features are derived by reconstructing the global feature Z into a three-dimensional tensor through a reverse projection operation, thereby preserving the spatial structure of the original matrix.

(15) $$Z^{\prime }=Reshape\left(W_{q}Z+b_{p}\right)\in R^{N\times M\times d}$$

Here, $W_{q}\in R^{N\times M\times d}$ denotes the reverse projection matrix. Subsequently, the relational feature vector at the indexed position $\left(i,j\right)$, denoted as $Z^{\prime }\left[i,j\right]\in R^{d}$, captures the specificity and association strength of the pair within the global relation matrix.

The two types of features are fused to construct a hybrid feature vector, formulated as follows:

(16) $$f_{ij}=\left.h_{pair}\right\|Z^{\prime }\left[i,j\right]\in R^{3d}$$

Deep Interaction Modeling: The hybrid feature vector $f_{ij}$ is input into a fully connected neural network to model nonlinear interactions. The network comprises two hidden layers followed by a Sigmoid output layer, ultimately generating the interaction probability.

Hidden Layer 1 extracts high-order features through the weight matrix $W_{1}\in R^{3d\times d_{h}}$ and bias term $b_{1}$.

(17) $$g_{ij}^{\left(1\right)}=ReLU\left(W_{1}^{T}f_{ij}+b_{1}\right)\in R^{d_{h}}$$

Hidden Layer 2 further compresses the feature dimensions, enhancing the nonlinear representation capability.

(18) $$g_{ij}^{\left(2\right)}=ReLU\left(W_{2}^{T}g_{ij}^{\left(1\right)}+b_{2}\right)\in R^{d_{h}/2}$$

Here, $W_{2}\in R^{d_{h}\times d_{h}/2}$.

The output layer generates the interaction probability through the Sigmoid function.

(19) $$p_{ij}=\sigma \left(W_{o}^{T}g_{ij}^{\left(2\right)}+b_{o}\right)\in \left(0,1\right)$$

Here, $W_{o}\in R^{d_{h}/2\times 1}$.

The model is optimized using the binary cross-entropy loss function to minimize the discrepancy between the predicted values and the true labels, thereby improving the model’s predictive accuracy.

### 2.3. Experiment

#### 2.3.1. Dataset

The herbal medicine data used in this study were derived from clinical case studies on hyperthyroidism, based on syndrome differentiation by Master of Traditional Chinese Medicine, Professor Zhou Zhongying. Efficacy data were obtained from the Chinese Pharmacopoeia (CHPA, 2015 edition), while ingredient and target data were obtained from publicly available databases including HERB [20], ETCM [21], and others.

Under the guidance of Professor Zhou Zhongying’s thyroid disease research team, a comprehensive dataset was compiled, comprising 128 core herbs, 139 efficacies, 2262 herb–target interactions, and 8298 herb–ingredient interactions relevant to hyperthyroidism treatment. By integrating relationships among herbs, efficacies, ingredients, and targets, we established 419 herb–efficacy relations, 14,302 herb–ingredient relations, and 7902 herb–target relations.

In the subsequent experiments, the dataset was randomly partitioned into training and testing sets at a ratio of 9:1.

#### 2.3.2. Evaluation Metrics

This study assesses model performance using six metrics: ppiuracy (ACC), Area Under the ROC Curve (AUC), Area Under the Precision-Recall Curve (AUPR), Precision, Recall, and F1-score. ACC quantifies overall prediction accuracy, with higher values indicating better reliability. AUC [22] assesses class differentiation, with higher values reflecting stronger discrimination. AUPR [23] evaluates performance on imbalanced datasets, where values closer to 1 indicate better detection of minority classes. Precision calculates the proportion of true positives to predicted positives, minimizing false positives, whereas Recall [24] measures the proportion of true positives among actual positives, minimizing false negatives. The F1-score provides a harmonic balance between Precision and Recall, capturing overall prediction accuracy. Collectively, these metrics offer a comprehensive assessment of MAMGN-HTI’s effectiveness in identifying positive herb–target interactions.

#### 2.3.3. Data Processing

For a given set of m herbs and n associated efficacies, each herb i can be represented by an efficacy vector $x_{i}=\left(y_{i},1,\cdots ,y_{i,j},\cdots ,y_{i,n}\right)$. Specifically, $y_{i,j}=1$ if efficacy j belongs to herb i; otherwise, $y_{i,j}=0$. Subsequently, the cosine value of each vector is computed, and pairwise cosine similarity is calculated to quantify the efficacy-based similarity between herbs a and b. Using the same methodology, additional similarity metrics are derived, including herb similarity based on efficacy, herb similarity based on targets, target pair similarity based on ingredients, and target pair similarity based on herbs.

The average values are computed for both the efficacy-based herb similarity and the target-based herb similarity. Similarly, the mean values are computed for the ingredient-based target pair similarity and the herb-based target pair similarity.

Based on the ingredient-derived herb pair similarity, a herb–herb network is constructed, in which nodes represent herbs and edges represent herb pairs sharing common efficacies (i.e., herb pairs with efficacy-based similarity greater than zero). To mitigate potential noise from low-similarity herb pairs, only the top α most similar neighboring herbs are retained for each herb, yielding a more robust herb network. Similarly, a target–target network is established based on target–ingredient similarity. To enhance the reliability of the target–target network, only the top β most similar neighboring targets with the highest similarity scores are preserved for each target.

#### 2.3.4. Parameter Settings

During the training of the MAMGN-HTI model, the Adam optimizer was selected after comparison with stochastic gradient descent (SGD) and others, where Adam consistently showed faster convergence and better validation performance. For hyperparameter tuning, learning rates of 1 × 10^−6^, 1 × 10^−5^, 1 × 10^−4^, and 5 × 10^−4^ were evaluated, with 1 × 10^−5^ identified as optimal, while the weight decay rate remained at 1 × 10^−5^. The node embedding dimension was set to 256, the output embedding dimension to 64, and the hidden layer size to 64 after evaluating alternatives ranging from 32 to 128. Model training was conducted with 10-fold cross-validation, each fold trained for 200 epochs. Binary cross-entropy loss was adopted to quantify the discrepancy between predicted probabilities and ground truth labels. To reduce model complexity, mitigate overfitting, and enhance performance, a pruning function was implemented. The dataset was partitioned into training and testing sets at a 9:1 ratio to ensure model robustness and generalization capability. In addition, an early stopping strategy was implemented, stopping training if the validation loss did not improve for 50 consecutive epochs, thereby preventing overfitting and improving reproducibility.

#### 2.3.5. Baselines

MAMGN-HTI was benchmarked against six state-of-the-art methods spanning two paradigms: topology-based and GNN-based approaches.

NEDTP [25] uses heterogeneous network embedding with random walk-based sampling to preserve topological information for drug–target interaction (DTI) prediction.

MultiDTI [26] combines network topology and molecular sequences using attention-based fusion to align chemical structures, target sequences, and pharmacological features in a shared space.

NeoDTI [27] utilizes graph convolutional layers for neural neighborhood aggregation, optimizing multi-hop feature integration via gradient descent.

IMCHGAN [28] employs hierarchical graph attention to prioritize key substructures, with matrix completion ensuring generalization to unseen drug–target pairs.

SGCL-DTI [29] integrates supervised contrastive learning with topology-semantic discrimination, exploiting edge perturbation and pharmacological similarity to select positive pairs.

EEG-DTI [30] combines shallow and deep graph convolutions with adaptive attention mechanisms, enhancing local–global context integration for DTI prediction.

## 3. Results

### 3.1. Experimental Results

**Figure 3:**
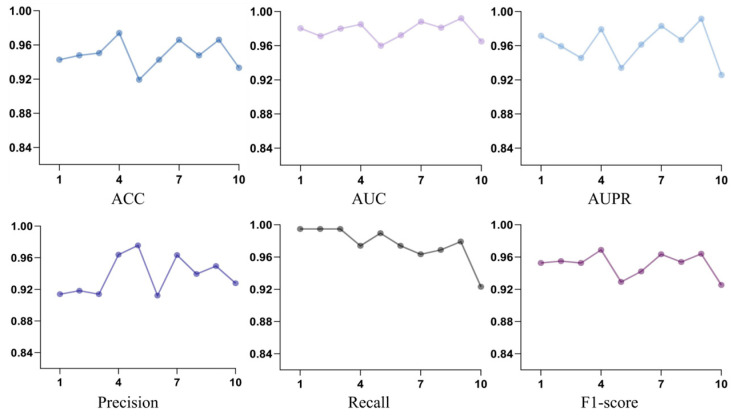
Performance results across different folds during ten-fold cross-validation on the constructed herb, efficacy, ingredient, and target datasets. The coloured lines indicate different evaluation metrics: the blue line represents ACC, the purple line represents AUC, the light blue line represents AUPR, the dark blue line represents Precision, the grey line represents Recall, and the dark purple line represents F1-score.

To assess model performance, ten-fold cross-validation was performed. As shown in Figure 3, the framework produced consistent results across all folds. The aggregated metrics were Accuracy of 0.9491, AUC of 0.9776, AUPR of 0.9618, Precision of 0.9278, Recall of 0.9756, and F1-score of 0.9507. The high AUC and AUPR values reflect strong discriminative capability and generalization performance, whereas Precision, Recall, and F1-score confirm the model’s reliability in predicting herb–target interactions.

### 3.2. Baseline Comparison Experiments

As shown in Table 1, MAMGN-HTI outperforms baseline models, demonstrating significant improvements in AUC and AUPR compared with similarity-based approaches. It achieves AUC gains of 0.0549 and 0.0186 and AUPR gains of 0.0006 and 0.0158 over NEDTP and MultiDTI, respectively. Its higher accuracy and F1-score further highlight its predictive strength. Compared with other GNN-based models, MAMGN-HTI excels, particularly in AUC and F1-score, outperforming all competing GNN approaches. All results include standard deviation metrics, reflecting the model’s stability and reproducibility across multiple iterations. These results confirm its effectiveness in capturing herb–target interactions, reinforcing its reliability for HTI prediction.

### 3.3. Ablation Experiment

To assess the contributions of key components in MAMGN-HTI, an ablation study was performed focusing on metapath selection, network structure, and skip connections. We evaluated the effects of removing four metapaths (HH, HIH, HTTH, HTITH), deactivating core networks (H-T, H-I, H-E, T-T), and eliminating ResGCN and DenseGCN.

Table 2 summarizes model performance under three ablation scenarios—metapath removal, network elimination, and residual connection deactivation—using AUC, AUPR, Precision, and F1-score. All results include standard deviation metrics, reflecting the model’s stability and reproducibility across multiple iterations. The results demonstrate that multi-scale metapaths, heterogeneous network architecture, and residual learning substantially improve prediction accuracy.

The complete MAMGN-HTI model achieves the highest performance, particularly in AUC and AUPR. The removal of metapaths substantially reduces predictive performance, underscoring their importance in capturing herb–target relationships. Ablation of network modules indicates that cross-modal interactions are critical, with Herb–Target and Target–Target networks exerting the most pronounced effects on AUC and Precision.

Analysis of residual connections reveals that removing ResGCN substantially reduces Precision and F1-score by impairing gradient propagation and feature integration. The removal of DenseGCN exerts a smaller effect, suggesting its secondary role in multi-scale feature aggregation. Eliminating both modules results in the largest performance decline, confirming their complementary contributions.

In summary, metapaths, network structure, and skip connections are critical for optimizing MAMGN-HTI, underscoring their pivotal role in enhancing model accuracy and node representation learning.

### 3.4. Generalization Ability Validation

To assess predictive performance, counterfactual reasoning was employed by concealing a subset of known interactions during training. The model was subsequently assessed based on its ability to infer these missing edges, minimizing overfitting. Predictions were classified as correct (matching actual relationships) or incorrect (misclassified edges), enhancing model optimization, particularly for non-existent interactions. To validate the effectiveness of the proposed method, five representative herbs were selected in this study: Salvia miltiorrhiza (Danshen), Glehnia littoralis (Beishashen), Coptis chinensis (Huanglian), Astragalus membranaceus (Huangqi), and Agastache rugosa (Peilan). Among the predicted results, 48 targets were correctly identified, whereas 2 were incorrect. The detailed results are provided in Table 3.

### 3.5. GO Enrichment Analysis

Key hyperthyroidism-related targets exhibited significant enrichment in the following biological processes: positive regulation of intracellular signal transduction (GO:1902533), positive regulation of cellular process (GO:0048522), positive regulation of cell proliferation (GO:0008284), regulation of apoptotic process (GO:0042981), and negative regulation of apoptosis (GO:0043066). These targets predominantly converge on three core pathological mechanisms: immune signal activation, dysregulation of metabolic homeostasis, and proliferation-apoptosis imbalance.

Analysis of three pivotal targets revealed their distinct pathological contributions: IL6 aberrantly activates NF-κB in thyrocytes, leading to MHC-II overexpression and promoting B-cell differentiation into antibody-secreting plasma cells, thereby sustaining thyroid-stimulating immunoglobulin (TSI) production. TSI directly stimulates thyrotropin receptors, exacerbating hormonal hypersecretion, while IL6-driven inflammation facilitates lymphocyte infiltration and thyroid tissue damage. Serum IL6 levels correlate with disease activity, reinforcing its role as a key mediator in Graves’ disease.

TP53 is functionally suppressed through MDM2 degradation or AKT inactivation, resulting in cell cycle dysregulation, BAX downregulation, and apoptosis resistance in thyrocytes. These alterations promote aberrant proliferation and genomic instability, with diminished TP53 expression in thyroid tissue correlating with goiter progression and therapeutic resistance.

AKT1 integrates TSHR/IGF1R signaling to activate thyroid peroxidase (TPO) and thyroglobulin (Tg), driving excessive hormone synthesis. It phosphorylates pro-apoptotic effectors (BAD, FOXO1) to enhance thyrocyte and orbital fibroblast survival, while mTORC1 hyperactivation accelerates metabolism, contributing to hypermetabolic phenotypes and insulin resistance. Elevated phospho-AKT in affected tissues underscore its central role in Graves’ orbitopathy pathogenesis.

### 3.6. Prediction Results

Based on the verification of the model’s generalization ability, its reliability was confirmed, enabling subsequent prediction experiments. The herb–target isomorphism relationship matrix obtained from the training experiments was used as input and mapped into a high-dimensional vector space through a linear projection layer. The two types of features were then integrated into a hybrid feature vector through a dual-way feature fusion mechanism. Nonlinear interactions were subsequently modeled using a fully connected network, and the final output was passed through an activation function to convert it into a probability value ranging from 0 to 1. For each herb–target pair, the predicted interaction probabilities were ranked in descending order, with higher-ranking pairs considered more likely to exhibit interactions.

**Figure 4:**
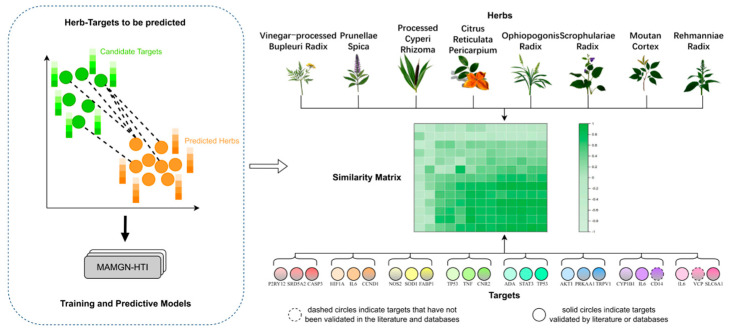
Performance results across different folds during ten-fold cross-validation on the constructed herb, efficacy, ingredient, and target datasets.

In this study, eight representative herbs were selected for validation: Vinegar-processed Bupleuri Radix (Cu Chaihu), Prunellae Spica (Xiakucao), Processed Cyperi Rhizoma (Zhi Xiangfu), Citrus Reticulata Pericarpium (Chenpi), Ophiopogonis Radix (Maidong), Scrophulariae Radix (Xuanshen), Moutan Cortex (Mudanhpi), and Rehmanniae Radix (Shengdi). The top three ranked herb–target pairs were validated through comprehensive literature and database searches, as illustrated in Figure 4. Performance results across different folds during ten-fold cross-validation on the constructed herb, efficacy, ingredient, and target datasets. Public databases, including ETCM (V2.0) [31], Herb (V2.0) [32], TCMSP [33], and existing relevant literature [34,35,36,37,38,39], were employed to verify the predicted associations between these eight herbs and their three predicted targets. Validation results indicated that a total of 21 targets were associated with the eight herbs. The effectiveness of the herbs and their predicted targets in the treatment of hyperthyroidism was assessed using the GeneCards [40] database. It was found that 11 of the targets have potential therapeutic effects on hyperthyroidism. The detailed validation results are shown in Table 4.

The experimental results of this study, validated through literature and database verification, are consistent with the predicted outcomes. Specifically, Prunellae Spica, Moutan Cortex, and Rehmanniae Radix were identified as collectively target IL6 in the treatment of hyperthyroidism, illustrating the multi-component and multi-target mechanisms underlying traditional Chinese medicine interventions. In addition to the verified herb–target interactions, the study also predicts that Ophiopogonis Radix may be associated with hyperthyroidism by acting on the TP53 target. This finding provides a basis for wet-lab validation, potentially reducing experimental costs and offering scientific support for TCM-based hyperthyroidism treatment.

## 4. Discussion

The proposed MAMGN-HTI model integrates metapath and attention mechanisms with cross-layer connection structures from ResGCN and DenseGCN, demonstrating robust performance in predicting herb–target interactions. By constructing a heterogeneous graph encompassing herbs, efficacies, ingredients, and targets, the model effectively captures the intricate relationships among diverse types of nodes. Leveraging metapath strategies and attention mechanisms, it systematically identifies key semantic paths, thereby enhancing the accuracy of herb–target interaction predictions.

The experimental results further validated the model’s effectiveness. For example, MAMGN-HTI predicted a potential association between Ophiopogonis Radix (Maidong) and hyperthyroidism through the target TP53, offering a concrete direction for subsequent wet-lab validation and potentially reducing experimental costs while improving research efficiency. Moreover, the study broadens the scope of candidate target identification for traditional Chinese medicine, facilitates the integration of classical TCM theories with modern computational approaches, and provides novel insights into the application of herbal medicine in the treatment of hyperthyroidism.

Despite the overall strong performance of the model, certain limitations remain. The prediction accuracy for complex or low-frequency herb–target relationships can still be improved. Although skip connections and attention mechanisms enhance feature propagation and representation, the model’s adaptability to heterogeneous graphs with diverse node types, as well as its generalization to other diseases and datasets, requires further optimization. Future work will address these challenges by incorporating more comprehensive heterogeneous node information, designing fine-grained graph structures, and integrating multi-task learning with dynamic attention mechanisms to strengthen predictive performance and generalization. In particular, the model will be extended and evaluated on additional diseases and datasets to systematically assess its robustness across diverse biological contexts. Beyond herbal medicine, the MAMGN-HTI framework will be explored for broader applications in systems characterized by complex interactions among multiple entity types. For instance, graph neural networks have been used to model topology, routing, and signal interference in communication networks [41], optimize circuit layout in VLSI global routing [42], and analyze social networks to identify influential nodes and predict information propagation [43]. These examples demonstrate the flexibility of heterogeneous graph and attention-based architectures, suggesting that the MAMGN-HTI framework could be adapted to support modeling of complex interactions in other biological and engineering systems.

## 5. Conclusions

This study introduces a graph neural network model, MAMGN-HTI, which integrates metapath and attention mechanisms to predict interactions between herbs and targets. By constructing a heterogeneous network representing traditional Chinese medicine and incorporating both ResGCN and DenseGCN architectures, the model effectively captures and fuses multi-level information of herb–target relationships, resulting in a significant enhancement in prediction performance.

The findings provide both empirical evidence and a theoretical framework for the application of traditional Chinese medicine in the treatment of hyperthyroidism. Beyond elucidating potential mechanisms of action for certain herbs, the study establishes new avenues for the modernization of TCM and the development of precision herbal medicine strategies. The proposed model demonstrates substantial potential for broad applications, including active compound screening and disease-related target discovery, thereby facilitating the deeper integration of traditional Chinese medicine with contemporary computational and biomedical technologies.

## Figures and Tables

**Table 1:** Comparison of MAMGN-HTI with the six baseline models on our dataset, with highest values in bold.

	Model	AUC	AUPR	Precision	F1-Score
Similarity-based	NEDTP	0.9227 (±0.0153)	0.9612 (±0.0227)	0.8928 (±0.0354)	0.8618 (±0.0259)
	MultiDTI	0.9590 (±0.0126)	0.9460 (±0.0253)	0.9263 (±0.0287)	0.8178 (±0.0304)
GNN-based	NeoDTI	0.9582 (±0.0149)	0.8745 (±0.0406)	0.8609 (±0.0423)	0.8218 (±0.0281)
	IMCHGAN	0.9705 (±0.0107)	0.8988 (±0.0352)	0.8974 (±0.0226)	0.8252 (±0.0273)
	SGCL-DTI	0.9240 (±0.0162)	0.9551 (±0.0218)	0.9185 (±0.0309)	0.9354 (±0.0156)
	EEG-DTI	0.9545 (±0.0134)	**0.9641** (±0.0185)	0.9162 (±0.0293)	0.8265 (±0.0277)
/	MAMGN-HTI	**0.9776 (±0.0096)**	0.9618 (±0.0202)	**0.9278 (±0.0257)**	**0.9507 (±0.0137)**

**Table 2:** The Ablation Effects of Different Metapaths, Network Structures, and Skip Connection Mechanisms on the MAMGN-HTI Model.

	Methods	AUC	AUPR	Precision	F1-Score
Metapath	HH	0.9621 (±0.0123)	0.9435 (±0.0235)	0.9012 (±0.0312)	0.9273 (±0.0187)
	HIH	0.9518 (±0.0137)	0.9304 (±0.0251)	0.8889 (±0.0330)	0.9124 (±0.0205)
	HTTH	0.9285 (±0.0162)	0.9012 (±0.0295)	0.8623 (±0.0367)	0.8856 (±0.0231)
	HTITH	0.9157 (±0.0175)	0.8826 (±0.0322)	0.8431 (±0.0385)	0.8682 (±0.0246)
Network	Herb–Target	0.9064 (±0.0181)	0.8723 (±0.0340)	0.8327 (±0.0410)	0.8589 (±0.0265)
	Herb–Ingredient	0.9639 (±0.0118)	0.9481 (±0.0228)	0.9127 (±0.0305)	0.9372 (±0.0178)
	Herb–Efficacy	0.9685 (±0.0109)	0.9532 (±0.0219)	0.9201 (±0.0280)	0.9445 (±0.0165)
	Target–Target	0.9352 (±0.0148)	0.9108 (±0.0275)	0.8745 (±0.0355)	0.8996 (±0.0212)
Skip_Connection	ResGCN	0.9283 (±0.0160)	0.9021 (±0.0298)	0.8614 (±0.0372)	0.8847 (±0.0230)
	DenseGCN	0.9527 (±0.0129)	0.9316 (±0.0249)	0.8912 (±0.0321)	0.9158 (±0.0192)
	ResGCN + DenseGCN	0.8992 (±0.0195)	0.8614 (±0.0360)	0.8216 (±0.0415)	0.8483 (±0.0280)
/	MAMGN-HTI_all	**0.9776 (±0.0096)**	**0.9618 (±0.0202)**	**0.9278 (±0.0257)**	**0.9507 (±0.0137)**

**Table 3:** Generalization Ability Validation Results of the Model for Five Herbal Medicines and Their Corresponding Targets.

Herb	Rank	Target	Result	Rank	Target	Result
Salvia miltiorrhiza	1	ACHE	T	6	EIF6	T
	2	CCND1	T	7	CES2	F
	3	CASP3	T	8	HERC5	T
	4	ADRB3	T	9	MAOB	T
	5	CYP1A2	T	10	CASP8	T
Glehnia littoralis	1	ASIC2	T	6	HIF1A	T
	2	ASIC1	T	7	ADK	F
	3	ACTB	T	8	AHCY	T
	4	ADA	T	9	PTGS2	T
	5	ADAR	T	10	PTGS1	T
Coptis chinensis	1	ADRA2A	T	6	CCND1	T
	2	ADRB1	T	7	PRSS1	T
	3	ADRB3	T	8	MAOB	T
	4	PTGS2	T	9	TNF	T
	5	PM20D2	T	10	TP53COR1	T
Astragalus membranaceus	1	PTGS1	T	6	KSR2	T
	2	RTP1	T	7	F8A1	T
	3	PRSS1	T	8	Topbp1-ps1	T
	4	RXRA	T	9	ICAM1	T
	5	ACP4	T	10	Ccpg1os	T
Agastache rugosa	1	CHRM1	T	6	PM20D2	T
	2	PTGS2	T	7	PTGS1	T
	3	SLC6A2	T	8	RXRA	T
	4	DHTKD1	T	9	BCL2	T
	5	ACP4	T	10	BAX	T

**Table 4:** Validation of the results of eight herbs against their corresponding targets, the fourth column shows the results of literature and database validation, and the fifth column shows the validation of the efficacy of the herbs against the predicted targets in the treatment of hyperthyroidism.

Herb	Rank	Target	Verify_source	Evidence
Vinegar-processed Bupleuri Radix	1	P2RY12	Li et al. [36]	—
	2	SRD5A2	ETCM (V2.0)	—
	3	CASP3	Herb (V2.0)	—
Prunellae Spica	1	HIF1A	ETCM (V2.0)	GeneCards
	2	IL6	Qin et al. [37]	GeneCards
	3	CCND1	Herb (V2.0)	—
Processed Cyperi Rhizoma	1	NOS2	TCMSP	—
	2	SOD1	Herb (V2.0)	GeneCards
	3	FABP1	ETCM (V2.0)	—
Citrus Reticulata Pericarpium	1	TP53	Huang et al. [34]	GeneCards
	2	TNF	Herb (V2.0)	GeneCards
	3	CNR2	ETCM (V2.0)	—
Ophiopogonis Radix	1	ADA	ETCM (V2.0)	GeneCards
	2	STAT3	Li et al. [35]	GeneCards
	3	TP53	—	GeneCards
Scrophulariae Radix	1	AKT1	Sheng et al. [38]	GeneCards
	2	PRKAA1	Herb (V2.0)	—
	3	TRPV1	ETCM (V2.0)	—
Moutan Cortex	1	CYP1B1	ETCM (V2.0)	—
	2	IL6	Herb (V2.0)	GeneCards
	3	CD14	—	—
Rehmanniae Radix	1	IL6	Herb (V2.0)	GeneCards
	2	VCP	—	—
	3	PLA2G1B	ETCM (V2.0)	—

## Data Availability

The ingredients and target data used in this study were obtained from the publicly available Herb database, accessed on 15 March 2025, through the website http://herb.ac.cn/. Herb data cannot be provided publicly due to privacy restrictions. Should you require access, please contact the corresponding author.

## Abbreviations

The following abbreviations are used in this manuscript:

TCM	Traditional Chinese Medicine
GNNs	Graph Neural Networks
HTI	Herb–Target Interaction
ResGCN	Residual Graph Convolutional Network
DenseGCN	Densely Connected Graph Convolutional Network
ACC	Accuracy
AUC	Area Under the Receiver Operating Characteristic Curve
AUPR	Area Under the Precision-Recall Curve
SGD	Stochastic Gradient Descent
GO	Gene Ontology
PPI	Protein–Protein Interaction
TSI	Thyroid-Stimulating Immunoglobulin
TPO	Thyroid Peroxidase
Tg	Thyroglobulin
NF-κB	Nuclear Factor kappa-light-chain-enhancer of activated B cells
mTORC1	Mechanistic Target of Rapamycin Complex 1
